# Multiple secondary cauda equina non-Hodgkin’s lymphoma: a case report and literature review

**DOI:** 10.1186/s12885-019-5800-4

**Published:** 2019-06-17

**Authors:** Yunchao Ban, Zhitao Jing, Jingyu Zou

**Affiliations:** Department of Neurosurgery, The First Affiliated Hospital of China Medical University, Nanjingbei street 155, Heping District, Shenyang, China

**Keywords:** Lymphoma, Non-Hodgkin, Cauda equine

## Abstract

**Background:**

Secondary central nervous system involvement of non-Hodgkin’s lymphoma (NHL) is rare and with poor prognosis, the most common pathological type is diffuse large B cell lymphoma (DLBCL). Although it can occur in any part of central nervous system, it rarely directly infiltrates the spinal cord or cauda equina.

**Case presentation:**

We present the case of 64-year-old immunocompetent man with a worsening pain of waist and left lower extremity, accompanied by numbness and paresis of bilateral lower extremity for 20 days. His previous medical history included a resection of painless mass in the left groin in another hospital 7 months ago, and the pathological diagnosis was non-Hodgkin small B cell lymphoma. Gd-enhanced MRI and F-18 FDG PET-CT scan demonstrated multiple infiltrations in the cauda equina. During the operation, we removed as many as 11 subdural-extramedullary bean-size lesions involving multiple nerve roots. The paralysis of his left leg recovered rapidly after the operation. During the follow-up period of more than one year, he underwent standard R-CHOP chemical therapy, no evidence of recurrence was noted until the 13th month, the patient died because of intracranial relapse.

**Conclusions:**

Imaging examination is important in the diagnosis of multiple secondary cauda equina non-Hodgkin’s lymphoma, and we highlight the significance of gadolinium-enhanced MRI and F-18 FDG-PET/CT in preoperative diagnosis as well as the previous history.

## Background

Central nervous system lymphoma (CNSL), including primary central nervous system lymphoma (PCNSL) and secondary central nervous system lymphoma (SCNSL) constitute 1–6% central nervous system malignant tumors [1, 2]. Up to 10% patients with non-Hodgkin’s lymphoma may progress CNS infiltrations with poor prognosis, and large diffuse B-cell lymphoma is the most common pathological type [3–6]. Although SCNSL could occur in any part of central nervous system, it mostly affects the brain, then leptomeninge, eyes and spinal cord. Primary or secondary lymphoma of cauda equina is extremely rare. In a large study of 150 cauda equina tumors the incidence of lymphomas was 1.3% [7] and over the years only a few case reports of primary cauda equina lymphoma have been published [8–10]. We present here a case of large diffuse B-cell lymphoma in the cauda equina, as well as a brief review the current literatures.

## Case presentation

A 64-year-old immunocompetent man presented to the outpatient clinic of Department of Neurosurgery, complained of a worsening pain of waist and left lower extremity, accompanied by numbness and paresis of bilateral lower extremity for 20 days. 7 months before admission, he took a biopsy of left groin mass, the pathological diagnosis was non-Hodgkin small B cell lymphoma. Immunohistochemical staining demonstrated the typical cells with CD5(+), CD20(−), Pax-5(+), Bcl-2(+), CD3(+), CD23(−), CyclinD-1(−), Ki-67(+ > 50%). According to the diagnosis, he underwent a standard CHOP chemical therapy immediately, and got a partial remission during the following 7 months, inguinal lymph nodes regressed by more than 50% and no new enlarged lymph node was detected by ultrasound examination. About 10 days after the last CHOP, he got a persistent pain in the waist and left thigh, accompanied by numbness and paresis, the symptoms had rapidly progressed to both lower extremity and left him wheelchair bound in 20 days, then he came to our department for further treament.

Physical examination demonstrated spastic paralysis of the left lower limb and hypesthesia of bilateral lower limb under L4 level, he also got tendon hyperreflexia and Babinski sign positive in the left side, with bladder dysfunction.

**Fig. 1:**
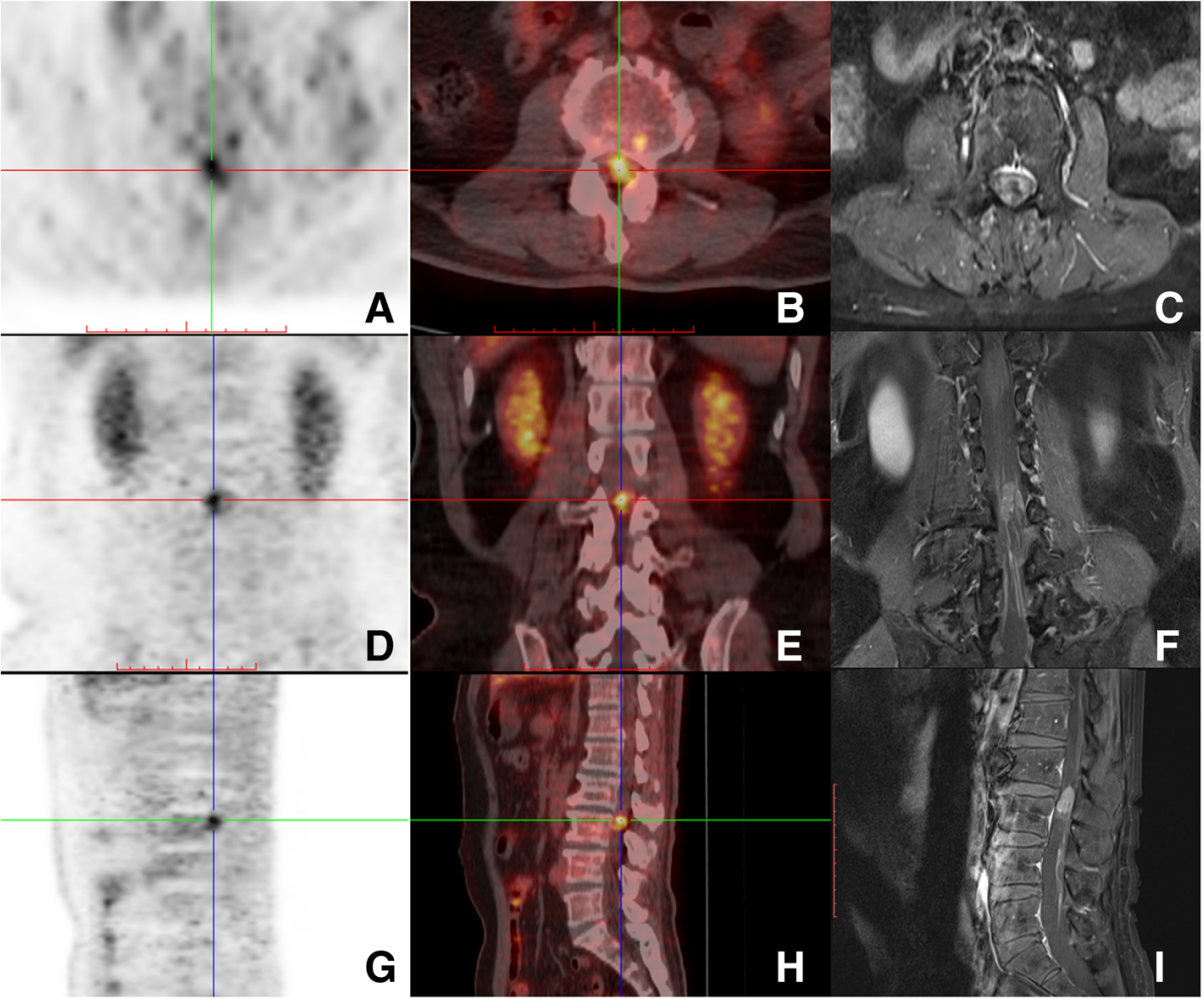
F-18 FDG-PET/CT and Gd-enhanced MRI images in axial, coronal and sagittal views. **a**,**b**,**d**,**e**,**g**,**h** show intrathecal high uptake of FDG at the L3 level. **c**,**f**,**i** show intrathecal mass of cauda equine at the L3 level with remarkable homogenous enhancement, several isolated modules can be distinguished in axial, coronal and sagittal views, cauda equina was compressed

Before admitted to our hospital, he took a whole-body F-18 FDG-PET/CT scan, which showed L3 level intrathecal FDG high uptake(Fig. 1a,b,d,e,g,h), without abnormal FDG uptake of other parts of central nervous system and the rest of the body, suggested probable involvement of lower spinal cord. Lumbar Gd-enhanced MRI showed L3 level multiple intrathecal lesions with isointense on T1WI and hypointense on T2WI, with remarkable homogenous enhancement. The total size of the lesions was about 2.29*1.39 cm with clear border, cauda equina was compressed badly. In the image of F-18 FDG-PET/CT, only 2 nodules could be distinguished, but in the high resolution MRI, especially T2WI image, we could distinguish as many as 7 (Fig. 1a-i).

**Fig. 2:**
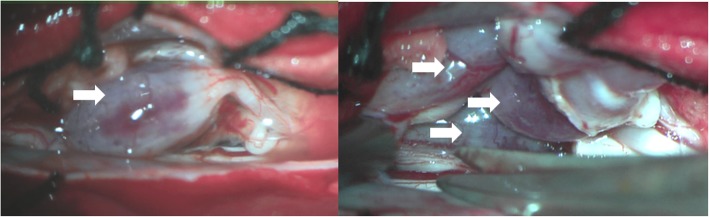
Images during the operation through microscope (Leica M525 F40, 8x) showed cauda equine roots enlargement, several isolated bean-size lesions with complete capsule involving nerve roots (arrows)

In consideration of the previous history, physical and image examination, we got a pre-operation diagnosis of secondary cauda equina lymphoma. A spinal canal decompression and tumor resection was performed 2 days after admission because the symptoms progressed rapidly, the patient developed complete paralysis and acute urinary retention. During the operation, we found the cauda equina was swollen and compressed badly, as many as 11 subdural-extramedullary bean-size nodules involving several nerve roots were found (Fig. 2). The nodules were red and with complete capsule, the relationship of tumors and cauda equina were too close to dissect, so we had to cut off the involved nerve roots to remove all the 11 nodules.

The paralysis of his left leg recovered rapidly since the operation, at the second day after operation, muscle strength was grade 2, and at the 7th day after operation, the patient could stand with assistance, only mild hypesthesia of the left leg remained. Post-operation MRI + C showed complete resection of the lesions with sufficient decompression. Pathological diagnosis was diffuse large B cell lymphoma, immunohistochemical stain showed CD20(+), Pax-5(+), CD3(+/−), Vimentin(+), NeuN(−), CD99(−),GFAP(−), S-100(−), Ki-67(+ > 50%), CK(−), CD56(−) Synaptophysin(−), TIF-1(−). The patient was transferred to the department of hematology at the 14th day after operation for further R-CHOP chemotherapy.

**Fig. 3:**
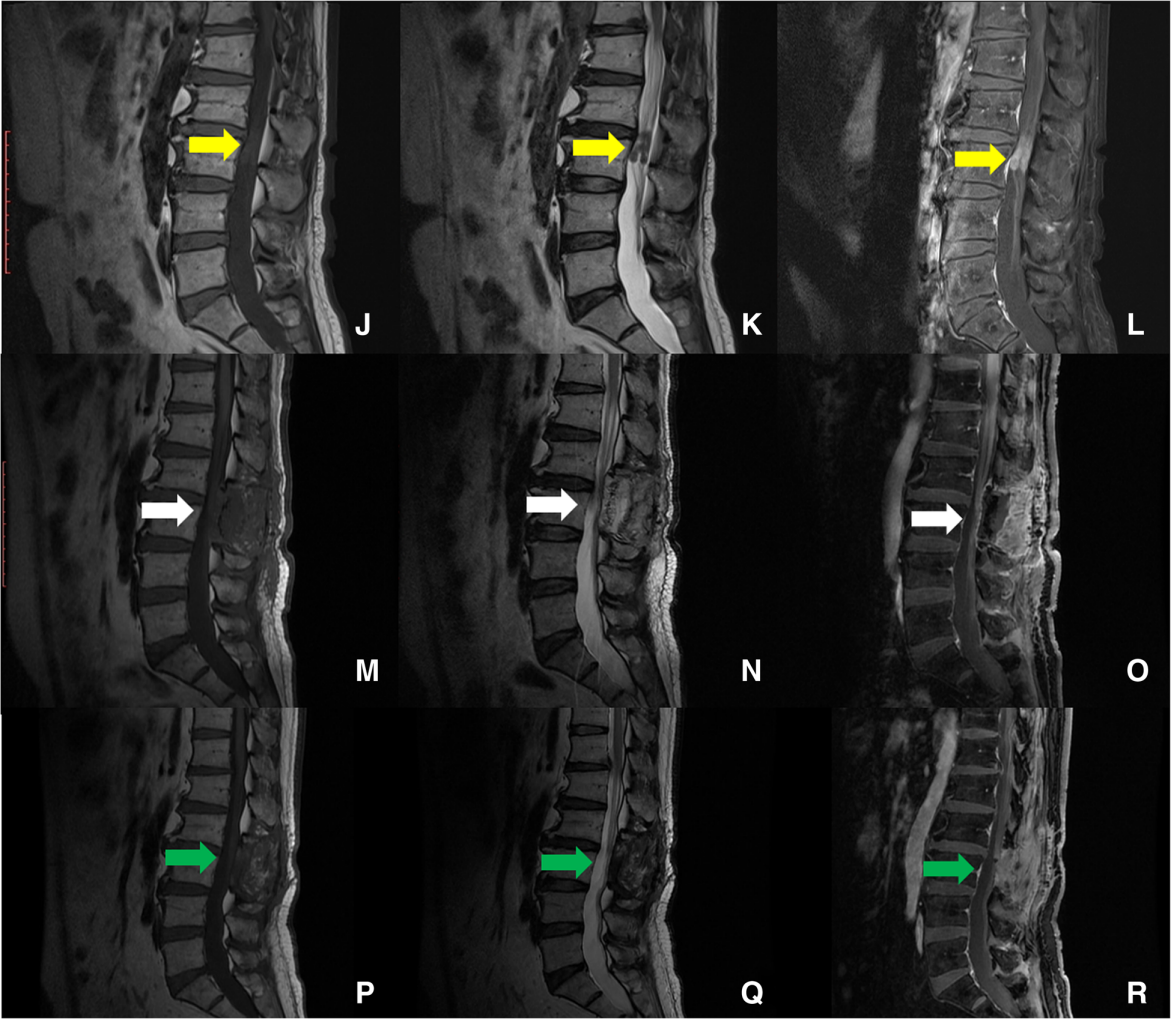
MRI images in sagittal sequence before operation showing isointense on T1WI (**j**) and hypointense on T2WI(**k**), with remarkable homogenous enhancement(**l**). 4 days (**m**,**n**,**o**)and 9 months(**p**,**q**,**r**) post-operation MRI showing totally resection of the tumors in corresponding sequences. The yellow arrows point to the tumor, the white and green arrows showed totally tumor resection with disappearance of enhancement

During the follow-up period of more than one year since the operation, the patient went on standard R-CHOP chemical therapy. He got a partial bladder function recovery at the 4th week after the operation, and could walk slowly without assistance by then, no new symptom of spinal cord was detected. A lumbar MRI + C at the 9th month showed no evidence of recurrence in situ (Fig. 3p-r). But at the 13th month, he visited the emergence room with severe headache and vomit, he was in such a bad condition that he was unable to stand MRI or F-18 FDG-PET/CT. Enhanced CT showed giant mass in bilateral frontal lobes with remarkable homogenous enhancement, circled by extensive brain edema, which obviously meant recurrence of the NHL. The patient and his family refused any further treatment, and finally he died in the following 2 weeks because of brain herniation.

## Conclusions

The incidence of central nervous system infiltration in system lymphoma varies between 10 and 20%, and indicating a poor prognosis [11]. Diffuse large B cell lymphoma is the most common type of non-Hodgkin lymphoma, and the incidence of its infiltration in central nervous system is about 1.1 to 10.4% [4, 12, 13]. A previous database study of 605 newly diagnosed DLBCL patients who had not undergone CNS prophylaxis showed that the probability of secondary CNS involvement at 1 year after diagnosis was 4.5% [14]. The prognosis of patients experiencing secondary CNS involvement, especially during progression or after relapse of systemic lymphoma is extremely poor [14, 15]. Spinal cord, especially cauda equina lymphoma is rarely reported.

In our case, the immunocompetent patient had normal lymphocyte count and fraction in peripheral blood, the diagnosis before operation was concluded from the previous biopsy history, symptoms, physical examination and radiography. He got a rapid progressing cauda equina syndrome in such a short time that we had to perform a total tumor resection instead of biopsy in hope of saving his motor ability, and fortunately his paralysis got recovery after the operation. Interestingly, the pathological diagnosis was non-Hodgkin small B cell lymphoma when he had the inguinal lymph node mass, but 7 months later, it transformed into diffuse large B cell lymphoma, that may suggest progressing of the disease. In the follow up period, the patient kept on standard R-CHOP treatment without any evidence of in situ relapse, until the 13th month he got a severe relapse in bilateral front lobes and eventually causing death. The course of the relapse was so short that we wondered if the malignancy grade of NHL progressed higher.

Flangan et al. [16] described the MRI feature of primary intramedullary spinal cord lymphoma, including multiple infiltration, enhancement, and cauda equina involvement. Suzuki et al. [17] summarized the MRI image findings of 23 cases of primary cauda equine lymphoma, the features of MRI are enlargement of the cauda equina with iso- or low intensity relative to the spinal cord signal on both T1WI and T2WI and the presence of enhancement of the cauda equina on contrast. In our case, lumbar Gd-enhanced MRI showed L3 level cauda equina enlargement, isolated lesions with isointense on T1WI and hypointense on T2WI and homogenous enhancement on contrast.

F-18 FDG-PET/CT plays an important role in monitoring and detecting relapses in malignant lymphoma patients, it is usually more sensitive than MRI in cranial nerves, nerve roots, and the cauda equina [18]. In our case, F-18 FDG-PET/CT excluded the possibility of involvement in other parts of the whole body especially CNS, so a surgical intervention could be considered; high resolution Gd-enhanced MRI demonstrated cauda equine roots involvement with severe compression in the L3 level, so we made a decision to resect all the tumors instead of biopsy. The resolution of MRI is much better than F-18 FDG-PET/CT, 7 of the lesions were detected in MRI vs only 2 in F-18 FDG-PET/CT. The intraoperative findings were consistent with the imaging performance, as many as 11 lesions were found and removed. According to our experience we recommend the combination of MRI and F-18 FDG-PET/CT in this kind of patients, it is effective not only for the diagnosis but also for surgery planning.

## Data Availability

The datasets used and analysed during the current study available from the corresponding author on reasonable request.

## Abbreviations

CNSL: Central nervous system lymphoma
DLBCL: Diffuse large B cell lymphoma
NHL: Non-Hodgkin’s lymphoma
PCNSL: Primary central nervous system lymphoma
SCNSL: Secondary central nervous system lymphoma
